# 

**Published:** 1883-04

**Authors:** 


### BOOKS AND PAMPHLETS RECEIVED.

AN INVESTIGATION INTO THE PARASITES IN THE PORK SUPPLY OF MONTREAL. By William Osler, M.D., M.R.C.P., and A. W. Clement. Montreal, 1883. Reprint from the Canada Medical and Surgical Journal, January, 1883.

ON CERTAIN PARASITES IN THE BLOOD OF A HOG. By William Osler, M.D. Reprint from Canadian Naturalist, Vol. 4, No. 7.

ON CANADIAN FRESH-WATER POLYZOA. By William Osler, M.D. Reprint from Canadian Naturalist, Vol 4, No. 7.

REMARKS ON CATTLE PLAGUE VACCINATION, with an appendix on the inoculation of goats with the virus of pleuro-pneumonia. Translated from the French of Dr. C. Pigeon. London, 1882.

THE PERCENTAGE OF COLLEGE-BRED MEN IN THE MEDICAL PROFESSION. By Charles McIntire, Jr., M.D. Read before the American Academy of Medicine, Philadelphia, 1883.

JAHRESBERICHT DER K. CENTRAL-THIERARZNEI-SCHULE IN MUNCHEN. 1881-1882. 8vo., 136 pages. Leipsig, 1883.

FIRST ANNUAL ANNOUNCEMENT of the School of Veterinary Medicine of Harvard University. 1883-84.

JOHNS HOPKINS UNIVERSITY CIRCULARS.

ANNALS ET BULLETIN de la Société de Medicine de Gand.

THE VETERINARIAN. A Monthly Journal of Veterinary Science. Edited by Professor Simonds. *London.*

THE QUARTERLY JOURNAL OF VETERINARY SCIENCE IN INDIA AND ARMY ANIMAL MANAGEMENT. Edited by Charles Steele, F.R.C.V.S., assisted by Fred. Smith, V.S., and John Henry Steel, V.S. *Bangalore, India.*

JOURNALS.—The Veterinary Journal, London. Der Zoologische Garten, Frankfort. Schweizerisches Archiv fur Thierheilkunde und Thierzucht, Bern, Archiv fur Wissenschaftliche und Practische Thierheilkunde, Berlin. Repertorium der Theirheilkunde, Stuttgart. Kansas City Review of Science and Industry. Chicago Medical Journal and Examiner. The Medical Record, New York. The College and Clinical Record, Philadelphia. Annals of Anatomy and Surgery, Brooklyn. New England Medical Monthly, Sandy Hook. Chicago Medical Review. Virginia Medical Monthly, Richmond. Nashville Journal of Medicine and Surgery. The Southern Clinic, Richmond. The Western Medical Reporter, Chicago. Journal of Cutaneous and Venereal Diseases, New York. Denver Medical Times. The St. Joseph Medical Herald. San Francisco Western Lancet. The Weekly Medical Review, Chicago and St. Louis. The Blacksmith and Wheelwright, New York. National Live Stock Journal, Chicago. The Breeder's Gazette, Chicago. Cultivator and Country Gentleman, Albany. Indiana Farmer, Indianapolis. Truth, San Francisco. Weekly Drover's Journal, Chicago. The Chicago Tribune. The Medical Register, Philadelphia. Spirit of the Times, New York.

